# Enhanced Gallium Extraction Using Silane-Modified Mesoporous Silica Synthesized from Coal Gasification Slag

**DOI:** 10.3390/molecules29225232

**Published:** 2024-11-05

**Authors:** Shiqiao Yang, Guixia Fan, Lukuan Ma, Chao Wei, Peng Li, Yijun Cao, Daoguang Teng

**Affiliations:** 1 School of Chemical Engineering, Zhengzhou University, Zhengzhou 450001, China; yangshiqiao0521@163.com (S.Y.); cumtfgx@126.com (G.F.); mlkhhh@stu.zzu.edu.cn (L.M.); zdhglipeng@zzu.edu.cn (P.L.); 2 Zhongyuan Critical Metals Laboratory, Zhengzhou University, Zhengzhou 450001, China; 3 The Key Lab of Critical Metals Minerals Supernormal Enrichment and Extraction, Ministry of Education, Zhengzhou 450001, China; 4 College of Chemical Engineering and Environment, China University of Petroleum (Beijing), Beijing 102249, China; chao_wei7997@163.com

**Keywords:** CGCS, cyclic acid leaching, silanol-modified mesoporous silica, gallium extraction

## Abstract

This study presents an innovative approach to utilize coal gasification coarse slag (CGCS) for efficient and low-cost gallium extraction. Using a one-step acid leaching process, mesoporous silica with a surface area of 258 m^2^/g and a pore volume of 0.15 cm^3^/g was synthesized. The properties of CGCS before and after acid leaching were characterized through SEM, FTIR, XRD, and BET analyses, with optimal conditions identified for maximizing specific surface area and generating saturated silanol groups. The prepared mesoporous silica demonstrated a 99% Ga(III) adsorption efficiency. Adsorption conditions were optimized, and adsorption kinetics, isotherms, and competitive adsorption behaviors were evaluated. Competitive adsorption with vanadium suggests potential application in Ga(III) extraction from vanadium-rich waste solutions. Furthermore, the recyclability of both the acid and adsorbent was explored, with the adsorbent maintaining over 85% adsorption efficiency after five cycles. The adsorption mechanism was further elucidated through SEM-EDS, XPS, and FTIR analyses. This work not only advances resource recovery from industrial waste but also offers a sustainable method for gallium extraction with industrial applications.

## 1. Introduction

Coal gasification slag (CGS) is a significant industrial waste byproduct in China, with improper disposal, such as stacking and landfilling, posing serious risks to the environment and human health [1,2,3,4,5]. Globally, vast amounts of CGS are stockpiled, and coal gasification coarse slag (CGCS) accounts for 60–80% of this waste [6,7]. Reusing this material to alleviate land occupation and mitigate environmental harm remains a challenge for industries [8]. Recently, the high silica (SiO_2_) content in CGS has attracted attention for its potential in ion adsorption, particularly in the adsorption of metals from dilute solutions. Despite this interest, the effective and economical utilization of SiO_2_ resources in CGCS for producing high-performance adsorbent materials remains an ongoing challenge, especially for the extraction of critical metals such as Ga(III), In(III), and Ge(IV) [9].

Mesoporous SiO_2_ has emerged as a promising material due to its large surface area [10], tunable pore size, and excellent chemical stability. These properties make it suitable for applications in catalysts [11], supports [11,12], adsorbents [13], sensors [14], and more [15]. Previous studies have demonstrated that CGS can be transformed into high-value mesoporous SiO_2_ through appropriate chemical and thermal treatments [16,17]. For instance, Xu et al. [18] prepared mesoporous SiO_2_ from coal gasification fine slag (CGFS) using acid leaching, calcination, and pH adjustment, resulting in a material with a larger surface area compared to commercially available SiO_2_. Similarly, Liu et al. [17] synthesized mesoporous glass microspheres with a surface area of 364 m^2^/g via simple acid leaching. Other researchers, such as Wei et al. [19], utilized CGFS to synthesize hierarchical porous SiO_2_ for CO_2_ adsorption, while Shu et al. [20] developed ZSM-5 molecular sieves from CGCS for volatile organic compound (VOC) adsorption. Chai et al. [21] used CGFS as a raw material, employing hierarchical synthesis to prepare SBA-15 and hierarchical X zeolite/carbon composite materials, modified with 1,3,5-trimethylbenzene and CO_2_ pre-activation to adjust their pore structure, followed by amino functionalization. Liu et al. [22] obtained novel functionalized nanostructured glass microspheres via foam flotation and simple acid leaching from CGFS, applied for the adsorption of Pb(II) and Congo Red. Yang et al. [23] modified CGCS using magnesium slag as a calcium source in the presence of NaOH to prepare a novel phosphate adsorbent. Gallium is commonly found in bauxite, lead–zinc ore, and coal mines [24]. Given that both coal gasification slag and gallium can originate from coal mines, this approach not only enables a more comprehensive utilization of coal resources but also reduces the likelihood of introducing additional impurity ions when using modified adsorbents derived from coal gasification slag to extract gallium from coal mines. In other words, modified materials from coal gasification slag are particularly suitable for gallium extraction. While these studies highlight the potential of CGS-derived materials, the adsorption of trace metals remains limited by the low density of surface functional groups, particularly hydroxyl (-OH) groups, which are crucial for enhancing adsorption capacity [24,25]. Adsorbents enriched with -OH groups have demonstrated superior performance in metal adsorption, making the development of such materials a pressing challenge [26].

To address these limitations, this study focuses on the acid leaching of CGCS to produce mesoporous silica with a surface enriched in silanol (-OH) groups, specifically for the adsorption of Ga(III). The effects of different acids (HCl, H_2_SO_4_, CH_3_COOH), acid concentrations, leaching times, and temperatures on the specific surface area and silanol group formation were investigated. As a result, mesoporous silica with a significantly enhanced surface area and silanol group content was synthesized. The adsorption behavior of Ga(III) was optimized, and the adsorption kinetics and isotherms were studied. Additionally, the recyclability of the acid and adsorbent was explored through cyclic regeneration experiments. Finally, the adsorption mechanism was elucidated through a detailed characterization. This approach not only enhances the high-value utilization of CGCS but also provides a cost-effective method for the industrial extraction of Ga(III), offering a sustainable solution for both waste management and critical metal recovery.

## 2. Results and Discussion

### 2.1. Optimization of Acid Leaching Conditions for Mesoporous Silica Construction

**Figure 1:**
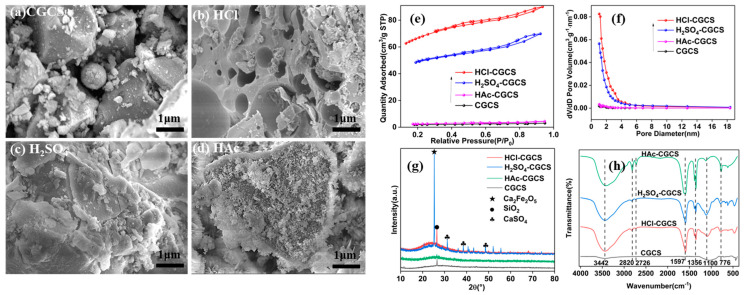
SEM images of (**a**) CGCS, (**b**) HCl-CGCS, (**c**) H_2_SO_4_-CGCS, and (**d**) HAc-CGCS, along with (**e**) adsorption–desorption isotherms, (**f**) pore size distribution, (**g**) XRD patterns, and (**h**) FTIR spectra.

Figure 1a–d shows the morphology of CGCS after modification with different acids. Untreated CGCS (Figure 1a) displays a compact morphology with spherical and block-like structures. Acid treatment, particularly with HCl, significantly increased the surface porosity of CGCS (Figure 1b). In contrast, H_2_SO_4_ and CH_3_COOH treatments (Figure 1c,d) resulted in fewer surface pores. The lower porosity in H_2_SO_4_-treated CGCS can be attributed to the potential precipitation of salts such as CaSO_4_, which block pores. Similarly, the weaker reactivity of CH₃COOH with metal oxides (Fe, Al, Ca) in CGCS limits its effectiveness in enhancing porosity [27]. These findings suggest that HCl is the most effective acid for modifying CGCS, significantly improving its performance.

Figure 1e–h presents the analysis of the specific surface area, composition, and functional groups of untreated and acid-treated CGCS samples. According to Figure 1e and Table 1, HCl-CGCS achieved the highest specific surface area of 240.05 m^2^/g—nearly 40 times greater than the untreated CGCS. H_2_SO_4_-CGCS showed a moderate increase, while CH_3_COOH-treated CGCS exhibited minimal improvement in surface area. Figure 1f shows that the pore sizes of all samples were predominantly between 2 and 4 nm, with HCl-CGCS and H_2_SO_4_-CGCS showing larger pore volumes than untreated CGCS, with HCl-treated CGCS performing slightly better. In contrast, CH_3_COOH-CGCS demonstrated a negligible improvement in pore volume.

Figure 1g presents the XRD patterns, where broad peaks between 20 and 30° indicate amorphous SiO_2_ [28]. Sharp peaks around 28° suggest crystalline SiO_2_, with HCl-CGCS and H_2_SO_4_-CGCS showing more prominent peaks compared to untreated CGCS, indicating the removal of metal oxide ash and an increased proportion of amorphous SiO_2_. CH_3_COOH-treated CGCS did not show a significant improvement in crystallinity over untreated CGCS, and the presence of Ca_2_Fe_2_O_5_ and CaSO_4_ peaks was more pronounced in H_2_SO_4_-treated CGCS.

Figure 1h shows FTIR spectra of the samples. The broad peak near 3442 cm^−1^ corresponds to O-H stretching vibrations, indicating the presence of bound water and surface hydroxyl groups formed during acid leaching [17,29,30,31]. The intensity of this peak increased significantly after HCl leaching, suggesting a higher density of surface silanol groups. Additional peaks at 1043 cm^−1^ correspond to Si-O anti-symmetric stretching, while the 776 cm^−1^ peak is associated with amorphous SiO_2_ [32]. The increased intensity of these peaks after HCl leaching confirms the successful removal of metal oxide ash and the enrichment of SiO_2_ content.

Thus, HCl was identified as the optimal acid for CGCS modification, warranting further investigation of the effects of acid concentration, leaching time, and temperature on mesoporous silica formation.

**Figure 2:**
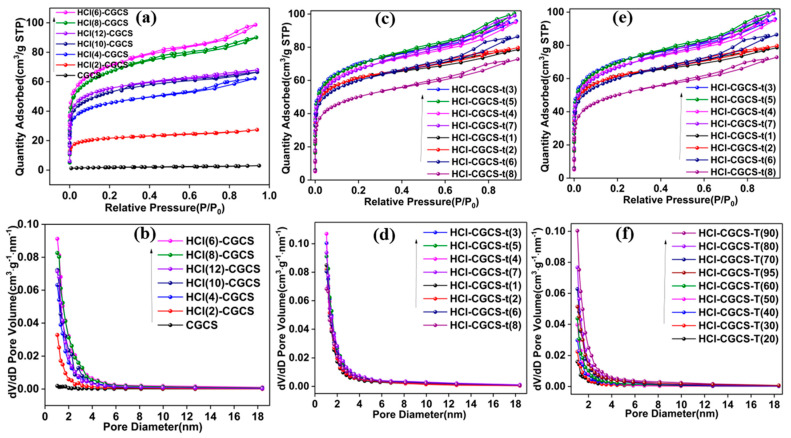
Isotherms of adsorption and desorption (**a**) and pore size distribution (**b**) of CGCS and HCl-CGCS with different acid concentrations; isotherms of adsorption and desorption (**c**) and pore size distribution curves (**d**) of HCl-CGCS with different acid immersion times; isotherms of adsorption and desorption (**e**) and pore size distribution curves (**f**) of HCl-CGCS with different acid immersion temperatures.

Figure 2a,b and Table 2 show the effect of varying HCl concentrations on the specific surface area and pore structure of CGCS. The surface area of HCl-treated CGCS initially increased with acid concentration, peaking at 6 mol/L HCl with a surface area of 258.40 m^2^/g (Figure 2b, Table 2). Beyond this concentration, the surface area declined slightly. Thus, 6 mol/L of HCl was selected as the optimal concentration for further experiments. Figure 2c,d and Table 3 illustrate the effect of immersion time on the specific surface area. The surface area of HCl-CGCS increased with time, reaching a maximum at 3 h (257.84 m^2^/g). Extending the leaching time beyond 3 h did not significantly affect the surface area or pore volume. Therefore, a leaching time of 3 h was chosen for subsequent experiments. Figure 2e,f and Table 4 demonstrate the influence of temperature on the specific surface area and pore structure. The surface area increased with temperature, reaching a maximum of 257.84 m^2^/g at 90 °C. Higher temperatures led to a slight decrease in surface area, indicating that 90 °C is the optimal temperature for acid treatment.

### 2.2. Influence of Acid Immersion on Silanol Group Formation

**Figure 3:**
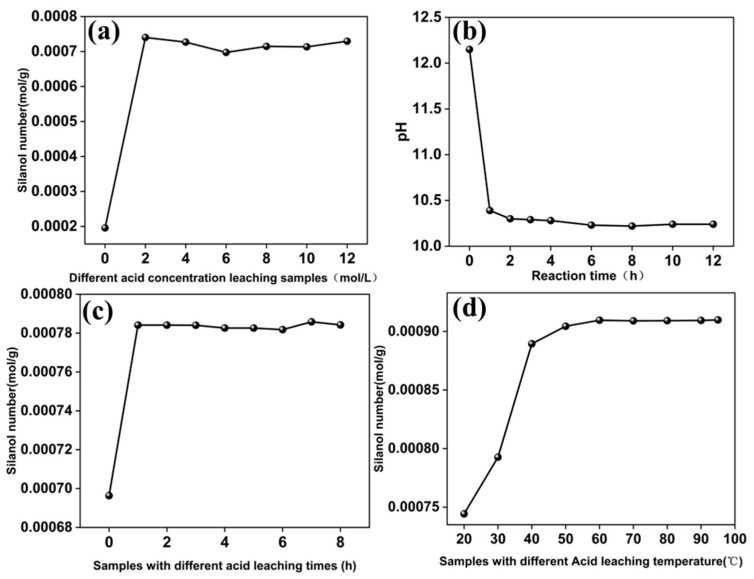
The reaction of HCl-CGCS with dilute NaOH, where the amount of consumed OH- approximately equals the number of silanol groups: (**a**) the number of silanol groups in HCl(C)-CGCS with different acid concentrations; (**b**) the relationship between the pH of the mixture of HCl(6)-CGCS and NaOH and the reaction time; (**c**) the number of silanol groups in HCl-CGCS-t(a) with different acid immersion times; (**d**) the number of silanol groups in HCl-CGCS-T(b) with different acid immersion temperatures.

Figure 3 presents the effects of HCl concentration, immersion time, and temperature on silanol group formation. Figure 3a shows that beyond 2 mol/L HCl, the quantity of silanol groups in HCl-treated CGCS remains relatively unchanged, indicating saturation. This suggests that concentrations of ≥2 mol/L are sufficient for saturating silanol groups in CGCS. Figure 3b shows that a reaction time of 2 h is optimal for detecting silanol groups, reducing the required time compared to the 12 h period reported in the literature [17]. Figure 3c indicates that an immersion time of ≥1 h saturates silanol group formation. Figure 3d demonstrates that temperatures of ≥60 °C are sufficient for saturating silanol groups with a concentration of 0.0009 mol/g.

### 2.3. Acid Immersion Mechanism

**Figure 4:**
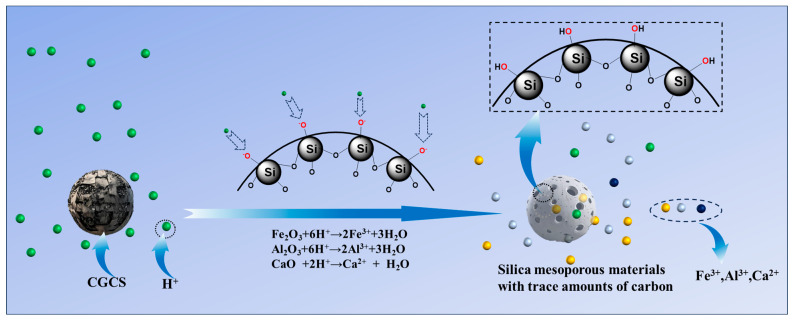
Acid immersion mechanism diagram.

The mechanism of HCl acid immersion in CGCS involves two key processes, namely (1) the leaching of ash content and (2) the formation of silanol groups (Figure 4). Acid leaching dissolves metal oxides, creating additional pores and increasing the specific surface area. Simultaneously, the high-temperature decomposition of CGCS breaks some Si-O bonds in SiO_2_, allowing oxygen atoms to bond with H⁺ ions, forming surface silanol groups (Si-OH). These processes are responsible for the enhanced adsorption capacity of HCl-treated CGCS.

### 2.4. Adsorption Behavior of Mesoporous Silica on Gallium

**Figure 5:**
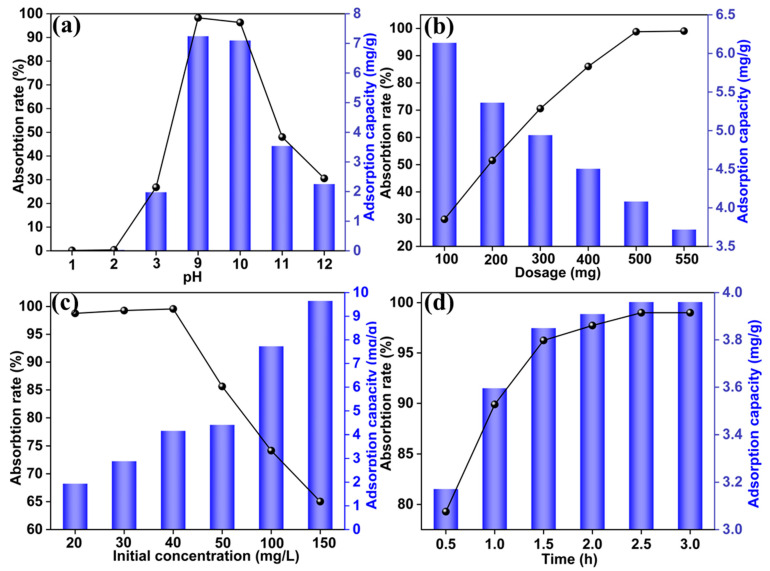
(**a**) Influence of pH, (**b**) adsorbent dosage, (**c**) initial concentration of Ga(III) solution, and (**d**) adsorption time on adsorption performance of HCl-CGCS.

Through a series of optimized experiments, a material labeled HCl(6)-CGCS-t(3)-T(90) (referred to as HCl-CGCS) was synthesized, with a specific surface area of 280 m^2^/g and saturated silanol groups. This material was tested for the adsorption of Ga(III). Figure 5 illustrates the static adsorption experiments and optimization of adsorption conditions for Ga(III) using HCl-CGCS. Figure 5a shows that the adsorption efficiency increases with pH, peaking at pH 9, where adsorption efficiency reaches approximately 100%. In Figure 5b, the adsorption performance increases with adsorbent dosage, stabilizing at 500 mg. Figure 5c demonstrates that as the initial concentration of Ga(III) increases, adsorption efficiency rises initially, peaking at 99% at a concentration of 40 mg/L before decreasing. Figure 5d shows that the adsorption rate increases up to 2.5 h, after which equilibrium is reached, with 99% adsorption efficiency.

The optimal adsorption parameters for Ga(III) were determined as pH 9, an adsorbent dosage of 500 mg, an initial Ga(III) concentration of 40 mg/L, and an adsorption time of 2.5 h. Under these optimized conditions, the adsorption efficiency of untreated CGCS was only 3.23%, while HCl-treated CGCS exhibited a 30-fold increase in Ga(III) adsorption efficiency.

**Figure 6:**
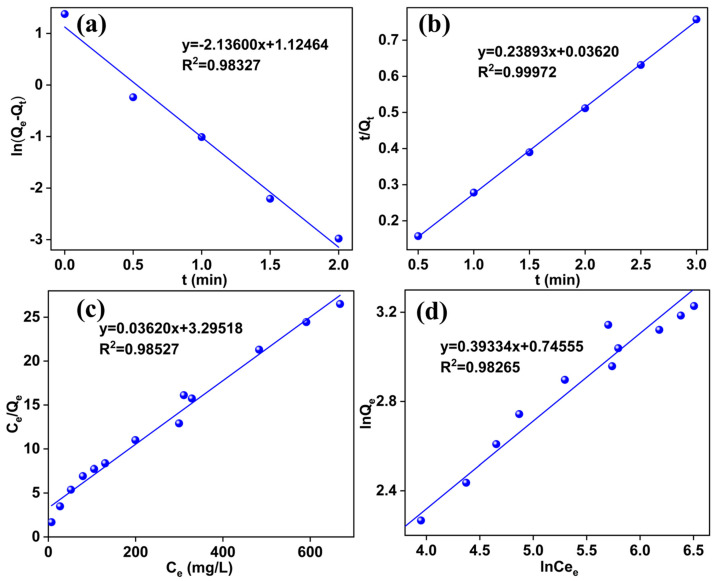
(**a**) Pseudo first-order kinetic model and (**b**) pseudo second-order kinetic model for Ga adsorption on HCl-CGCS and (**c**) Langmuir and (**d**) Freundlich isotherm fitting curves.

Table 5 presents the kinetic parameters derived from pseudo first-order and pseudo second-order models, shown in Figure 6a,b. Both models show R^2^ values above 0.95, indicating their suitability in describing the adsorption process. However, the pseudo second-order model fits the data more closely, suggesting that chemical adsorption predominates during Ga(III) adsorption.

Table 6 lists the isotherm fitting parameters for the Langmuir and Freundlich models. Both models exhibit high *R*^2^ values, indicating their effectiveness in describing the Ga(III) adsorption process. However, the Langmuir model provides a slightly better fit, suggesting that monolayer adsorption is more significant. The Langmuir model’s dimensionless constant (*R_L_*) values for Ga(III) are between 0 and 1, indicating favorable adsorption conditions.

### 2.5. Competitive Adsorption

In the competitive adsorption experiment, we investigated Ga-Al competitive adsorption, and the results indicated that the adsorbent does not exhibit selectivity between Ga and Al. Therefore, we focused on discussing the Ga-V competition experiment.

The selective recovery of gallium from vanadium-containing waste solutions is of significant industrial interest [33]. Figure S1 (Supplementary Materials) illustrates the competitive adsorption behavior of Ga(III) and V(V) on HCl-CGCS. The results show that HCl-CGCS exhibits significantly higher adsorption for Ga(III) compared to V(V). A selectivity coefficient (*Sel_Ga/V_*) greater than one (Sel = 1.5736), as shown in Table S1, confirms the preferential adsorption of Ga(III) from Ga-V binary systems.

### 2.6. Regeneration Cycle

The acid regeneration and adsorption recycling processes are crucial for the economic and environmental sustainability of this approach. This experiment evaluated the recycling potential of HCl and the adsorbent.

**Figure 7:**
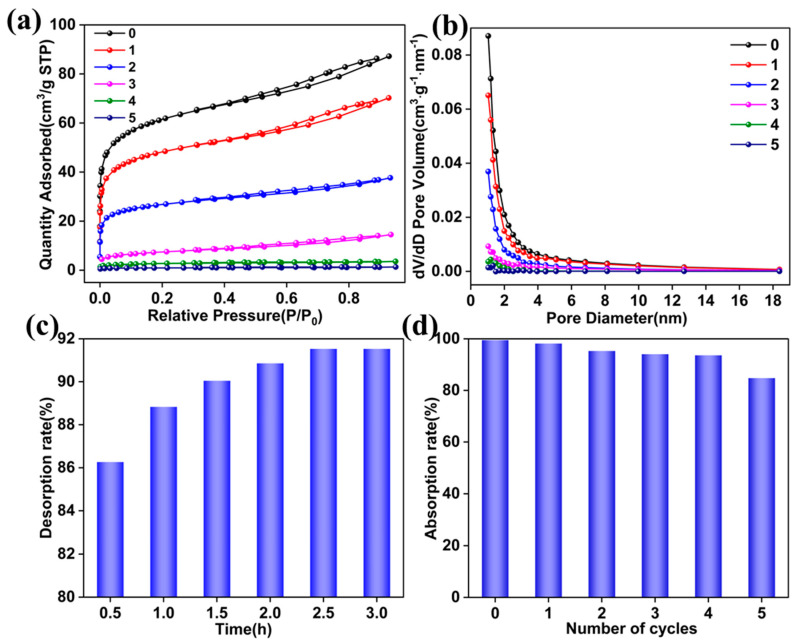
(**a**) Adsorption-desorption isotherms of HCl-CGCS after 5 cycles. (**b**) Pore size distribution. (**c**) Effect of HCl solution elution time on desorption efficiency. (**d**) Adsorption performance after 5 cycles.

Figure 7a,b and Table 7 show that cycling 6 mol/L HCl (30 mL) for five cycles with 4 g of CGCS results in a gradual decline in surface area and pore volume, while pore size remains largely unchanged. After the first cycle, the surface area remained above 200 m^2^/g. Even after four cycles, the surface area remained higher than that of untreated CGCS, indicating that HCl recycling retains its positive effect on CGCS modification. The repeated use of HCl four times demonstrates an efficient green cycling approach for enriching metal ions and utilizing residual ash content.

As shown in Figure 7c, during the desorption process, 50 mL of 0.15 mol/L HCl achieved a desorption rate of 91.53% in 2.5 h. The eluate contained Si, Al, Fe, and Ca at concentrations ≤ 5 ppm, indicating effective desorption. Figure 7d shows that the adsorption efficiency of HCl-CGCS remained above 85% after five cycles, confirming the adsorbent’s excellent cyclic regeneration performance.

### 2.7. Adsorption Mechanism Exploration

**Figure 8:**
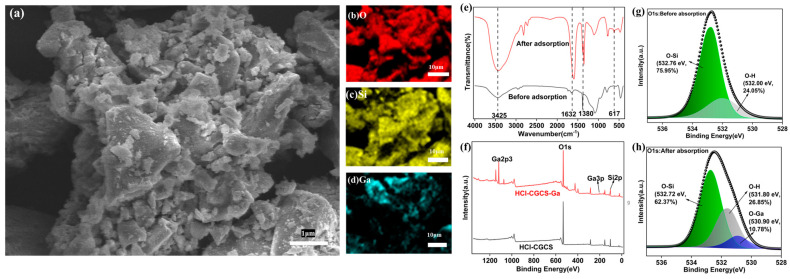
SEM Images of HCl-CGCS after Ga(III) adsorption (**a**); elemental distribution EDS maps of (**b**) O, (**c**) Si, and (**d**) Ga; FTIR spectra before and after Ga adsorption (**e**); XPS survey spectrum (**f**); O1s fine spectrum of HCl-CGCS before Ga adsorption (**g**); and O1s fine spectrum of HCl-CGCS after Ga adsorption (**h**).

The mechanism of Ga(III) adsorption on HCl-CGCS was further investigated using energy-dispersive X-ray spectroscopy (EDS), FTIR, and XPS analyses. Figure 8 shows significant Ga signals on HCl-CGCS after adsorption, confirming Ga(III) adsorption on the surface.

FTIR spectra (Figure 8e) reveal peaks at 3425 cm^−1^ and 1632 cm^−1^, corresponding to H_2_O and -OH vibrations, respectively [17,29,30]. These peaks intensify after Ga(III) adsorption, suggesting the adsorption of Ga(OH)₄^−^ species. A new peak at 617 cm^−1^ after adsorption corresponds to O-Ga bonds [34]. The XPS analysis (Figure 8f) reveals Ga3p1 and Ga2p3 peaks after adsorption, confirming the coordination of Ga(III) with the adsorbent. A comparison of the O1s fine spectra before and after adsorption (Figure 8g,h) shows an increase in O-H signal intensity and the appearance of an O-Ga peak at 530.9 eV [35], further supporting the formation of O-Ga bonds through electrostatic and chemical interactions.

**Figure 9:**
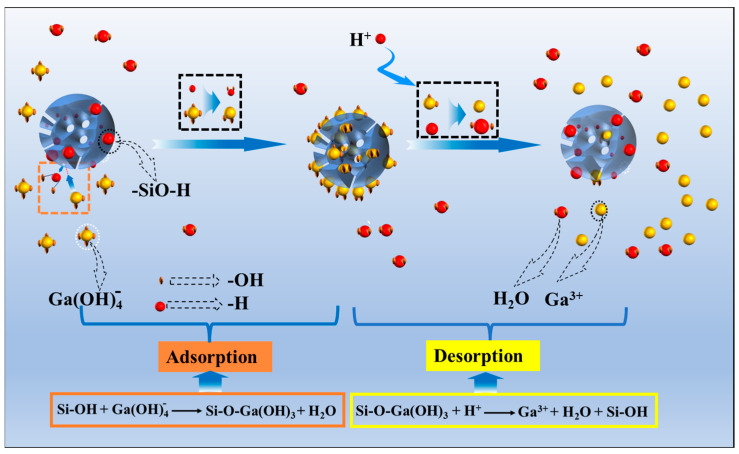
Chemical adsorption and desorption mechanism of Ga(OH${)}_{4}^{-}$.

Figure 9 presents the proposed chemical adsorption and desorption mechanism. During adsorption, Si-OH groups ionize, releasing H⁺ ions that combine with Ga(OH)_4_^−^ to form H_2_O. Simultaneously, Si-O^−^ coordinates with Ga in Ga(OH)_3_.

## 3. Experimental Section

The acid leaching and adsorption experiments are detailed in the Supporting Information.

### 3.1. Materials and Reagents

CGCS used in this study was sourced from Shenhua Group, Ningxia, China, produced in a modified Siemens (GSP) gasifier. The chemical composition of CGCS was determined by X-ray fluorescence (XRF) and industrial analysis, as summarized in Table 8. The raw CGCS was crushed to a particle size of approximately 200 mesh using a grinder, and the resulting powder was used in all subsequent experiments. Hydrochloric acid (HCl, 37%), sulfuric acid (H_2_SO_4_, 98%), and acetic acid (CH_3_COOH, 99.5%) were obtained from Luoyang Chemical Reagent Factory (Henan, China). Laboratory-grade ultrapure water was used throughout the experiments.

### 3.2. Characterization

The phase composition and crystalline structure of the materials were analyzed using X-ray diffraction (XRD, D8-ADVANCE, Bruker, Karlsruhe, Germany) with a scanning angle range from 10° to 80°. Surface functional groups were characterized using Fourier transform infrared spectroscopy (FTIR, FT-IR200, Shimadzu, Kyoto, Japan), with samples prepared by the KBr pellet method and scanned in the 4000–400 cm^−1^ infrared spectrum range. Microstructural observations and elemental distributions were examined using scanning electron microscopy with energy-dispersive X-ray spectroscopy (SEM-EDS, ZEISS Sigma 300, ZEISS, Oberkochen, Germany). Specific surface area, pore size distribution, and pore structure parameters were determined using the Brunauer–Emmett–Teller (BET) method (BELSORP-Max 11, MicrotracBEL, Osaka, Japan) after sample pretreatment at 300 °C for 4 h. The data were processed using BELSORP-Max software (BELSORP Analysis Software V5.4.1). The ion concentrations in solutions were quantified by inductively coupled plasma optical emission spectroscopy (ICP-OES, 5800 ICP-OES, Agilent Technologies, Penang, Malaysia). Surface elemental composition and valence states were analyzed via X-ray photoelectron spectroscopy (XPS, Thermo Scientific K-Alpha, Wilmington, NC, USA).

### 3.3. Silanol Group Quantification

Identifying silanol groups through infrared detection is challenging due to the overlap with the water absorption peak. Therefore, an acid–base titration method [17,36,37] was employed to quantify silanol groups [38,39,40], with the amount of NaOH consumed corresponding to the number of silanol groups. This method was adapted for a quantitative analysis of silanol groups in HCl acid-leached CGCS under varying acid concentrations, times, and temperatures, and the equilibrium reaction time between HCl-treated CGCS and NaOH was determined.

The procedure was as follows: 1 g of HCl(C)-CGCS-t(8)-T(90) was added to 50 mL of 0.02 mol/L NaOH solution and stirred magnetically at room temperature for 12 h. The initial pH (pH_0_) and final pH after 12 h (pH_12_) were measured using a pH meter (PHS-3E). The effect of acid concentration on silanol formation was determined based on the change in pH. Additionally, the pH of the reaction solution between HCl-treated CGCS and NaOH was measured at regular intervals to determine the reaction equilibrium time (t). The effects of acid impregnation time and temperature on silanol formation were also investigated under optimized conditions. The surface hydroxyl content (C) was calculated using the following equations:

NaOH (50 mL, 0.02 mol/L) + CGCS-OH (1 g) → CGCS-ONa + H_2_O (1)

*C*_0_ = 10^−14 *+ pH*^_0_ (2)

*C_t_* = 10^−14 *+ pH*^*_t_* (3)

*C* = *C*_0_ − *C_t_* (4)

where *C*_0_ and *C_t_* represent the initial and final OH⁻ concentrations in the solution, respectively, and *C* is the surface hydroxyl content of HCl-treated CGCS (mol/L).

Note: A(C)-CGCS-t(a)-T(b): A is the acid used for leaching CGCS, C is the acid concentration, a is the leaching time, and b is the leaching temperature.

### 3.4. Cycle Experiment

To evaluate acid recyclability, HCl (6 mol/L, 30 mL) was mixed with 4 g of CGCS in a pressure-resistant bottle, and the mixture was subjected to acid leaching in a constant temperature water bath shaker at 90 °C and 200 rpm for 3 h. The filtrate from this reaction was reused to leach another 4 g of CGCS under the same conditions, and the process was repeated for five cycles.

For the adsorption-desorption-readsorption cycle, CGCS-derived mesoporous silica was used to adsorb Ga(III) under optimal conditions. After adsorption, the adsorbent was desorbed using HCl (0.15 mol/L, 50 mL) for 3 h. The Ga, Fe, Al, and Ca contents in the solution at desorption equilibrium were measured by ICP-OES. The desorption equilibrium time, desorption rate, and impurity ion concentration were determined. The desorption rate was calculated similarly to the adsorption rate.

## 4. Conclusions

This study demonstrates the economic, environmental, and sustainable utilization of coal gasification coarse slag (CGCS) for the extraction of the critical rare metal gallium using acid leaching methods. The key findings of this research are summarized as follows:

Preparation of mesoporous silica via HCl leaching: CGCS was effectively transformed into mesoporous silica with a high specific surface area by leaching with 6 mol/L HCl at 90 °C for 3 h. The resulting mesoporous silica exhibited a specific surface area of 258 m^2^/g, representing a 40-fold increase compared to untreated CGCS. The saturation of silanol groups was achieved with HCl concentrations of ≥2 mol/L, leaching times of ≥1 h, and temperatures of ≥60 °C.

Outstanding adsorption performance and selectivity for Ga(III): Under optimized conditions (500 mg of mesoporous silica, 50 mL of 40 mg/L Ga(III) solution, pH 9, at 30 °C for 2.5 h), the material achieved 99% adsorption efficiency. In contrast, untreated CGCS under the same conditions adsorbed only 3.23%, indicating a 30-fold improvement in adsorption capacity. The adsorption process followed pseudo second-order kinetics, with the Langmuir model describing the isotherms, suggesting a combination of physical and chemical adsorption mechanisms. Additionally, the material demonstrated a selective recovery of Ga in a Ga-V binary system with a selectivity coefficient (*Sel*) of 1.5736.

Cyclic regeneration of HCl solution and mesoporous silica: The leaching of 4 g of CGCS with 6 mol/L HCl retained a specific surface area of 202.22 m^2^/g after one cycle, showcasing the potential for resource enrichment and a hierarchical utilization of the material. Adsorption experiments over five cycles showed a consistent Ga(III) adsorption efficiency above 85%, demonstrating the effective recyclability of both the HCl solution and the mesoporous silica.

Adsorption and desorption mechanism: The adsorption mechanism involves the dissociation of silanol groups (Si-OH), with H^+^ ions reacting with Ga(OH)_4_^−^ to form H_2_O, while Si-O^−^ coordinates with Ga(OH)_3_, forming Si-O-Ga(OH)_3_. During desorption, H^+^ ions attack the Si-O-Ga(OH)_3_ complex, releasing Ga^3+^, H₂O, and regenerating Si-OH. The reaction can be described by the following equations:

Si-OH + Ga(OH)_4_^−^ → Si-O-Ga(OH)_3_ + H_2_O

Si-O-Ga(OH)_3_ + H^+^ → Ga^3+^ + H_2_O + Si-OH

These findings provide a promising approach for the sustainable extraction of Ga(III) from industrial waste, with potential applications in resource recovery and environmental remediation.

## Figures and Tables

**Table 1:** Pore structure parameters of CGCS and acid-treated samples.

Sample	a_s,BET_ (m^2^/g)	Total Pore Volume (cm^3^/g)	Average Pore Diameter (nm)
CGCS	6.3855	0.0047	2.9184
HCl-CGCS	240.0500	0.1393	2.3217
H_2_SO_4_-CGCS	179.3500	0.1081	2.4100
HAc-CGCS	8.2362	0.0068	3.3101

**Table 2:** Pore structure parameters of CGCS and HCl-CGCS with different acid concentrations.

Sample	a_s,BET_ (m^2^/g)	Total Pore Volume (cm^3^/g)	Average Pore Diameter (nm)
CGCS	6.3855	0.0047	2.9184
HCl(2)-CGCS	79.7940	0.0424	2.1235
HCl(4)-CGCS	167.7100	0.0963	2.2967
HCl(6)-CGCS	258.4000	0.1526	2.3626
HCl(8)-CGCS	240.0500	0.1393	2.3217
HCl(10)-CGCS	194.9600	0.1029	2.1116
HCl(12)-CGCS	204.4900	0.1052	2.0570

**Table 3:** Pore structure parameters of HCl-CGCS with different acid immersion times.

t (h)	a_s_,_BET_ (m^2^/g)	Total Pore Volume (cm^3^/g)	Average Pore Diameter (nm)
t = 1	227.7500	0.1216	2.1364
t = 2	227.4900	0.1234	2.1694
t = 3	257.8400	0.1482	2.2987
t = 4	247.4413	0.1471	2.3786
t = 5	253.4111	0.1553	2.4513
t = 6	221.3612	0.1338	2.4172
t = 7	245.4923	0.1533	2.4972
t = 8	182.1936	0.1127	2.4737

**Table 4:** Pore structure parameters of HCl-CGCS with different acid immersion temperatures.

T (°C)	a_s_,_BET_ (m^2^/g)	Total Pore Volume (cm^3^/g)	Average Pore Diameter (nm)
T = 20	43.5290	0.0244	2.2414
T = 30	61.2870	0.0318	2.0733
T = 40	87.2240	0.0455	2.0885
T = 50	117.5300	0.0588	2.0016
T = 60	115.7700	0.0617	2.1332
T = 70	172.7800	0.0915	2.1191
T = 80	199.8900	0.1111	2.2237
T = 90	257.8400	0.1482	2.2987
T = 95	248.2500	0.0920	2.4819

**Table 5:** Kinetic parameters for Ga(III) adsorption on HCl-CGCS.

*Q_e,exp_*, (mg/g)	Pseudo First-Order			Pseudo Second-Order		
	*Q_e_* (mg/g)	K_1_ (min^−1^)	*R* ^2^	*Q_e_* (mg/g)	K_2_ (g·mg^−1^·min^−1^)	*R* ^2^
3.9600	3.0791	2.1360	0.9833	4.1853	1.5770	0.9997

**Table 6:** Isotherm fitting parameters for Ga(III) adsorption on HCl-CGCS.

*T* (K)	*Q_m,_*_exp_ (mg/g)	Langmuir Model				Freundlich Model		
		*Q_m_* (mg/g)	b (L/mg)	*R* ^2^	*R_L_*	*n*	*K_F_* (mg/g)(L/mg) ^1/n^	*R* ^2^
303.1500	27.6243	0.0110	0.9853	0.9853	0.6947	2.5423	2.1076	0.9827

**Table 7:** Pore structure parameters of CGCS after 5 cycles of HCl cyclic acid immersion.

Number of Cycles	a_s_,_BET_ (m^2^/g)	Total Pore Volume (cm^3^/g)	Average Pore Diameter (nm)
0	257.0100	0.1349	2.3609
1	202.2232	0.1087	2.4264
2	113.0098	0.0582	2.3269
3	30.84711	0.0225	3.2899
4	10.0210	0.0055	2.2059
5	3.4663	0.0020	2.3490

**Table 8:** Chemical composition of CGCS (wt%).

Component	SiO_2_	Fe_2_O_3_	Al_2_O_3_	CaO	MgO	K_2_O	Na_2_O	TiO_2_	LOI	Others
Content (%)	50.24	14.72	14.75	10.46	2.27	2.26	1.79	1.00	1.32	1.19

## Data Availability

The original contributions presented in the study are included in the article/Supplementary Materials; further inquiries can be directed to the corresponding authors.

## Supplementary Materials

The following supporting information can be downloaded at: https://www.mdpi.com/article/10.3390/molecules29225232/s1, Figure S1: Competitive adsorption in the Ga-V binary system; Table S1: Selectivity coefficients for Ga/V competitive adsorption.

## References

[1] Qu J., Zhang J., Li H., Li S., Hou X., Chang R., Zhang Y. (2024). Coal gasification slag-derived highly reactive silica for high modulus sodium silicate synthesis: Process and mechanism. Chem. Eng. J..

[2] Gai H., Feng Y., Lin K., Guo K., Xiao M., Song H., Chen X., Zhou H. (2017). Heat integration of phenols and ammonia recovery process for the treatment of coal gasification wastewater. Chem. Eng. J..

[3] Gary F. (2006). Bennett. Book review: Gasification technologies: A primer for engineers and scientists. J. Hazard. Mater..

[4] Hsieh P.Y., Kwong K., Bennett J. (2016). Correlation between the critical viscosity and ash fusion temperatures of coal gasifier ashes. Fuel Process. Technol..

[5] Jia W., Guo Y., Guo F., Li H., Li Y., Zhang Y., Wu J., Si C. (2023). Co-combustion of carbon-rich fraction from coal gasification fine slag and biochar: Gas emission, ash sintering, heavy metals evolutions and environmental risk evaluation. Chem. Eng. J..

[6] Qu J., Zhang J., Li H., Li S. (2021). A high value utilization process for coal gasification slag: Preparation of high modulus sodium silicate by mechano-chemical synergistic activation. Sci. Total Environ..

[7] Su S., Tahir M.H., Cheng X., Zhang J. (2024). Modification and resource utilization of coal gasification slag-based material: A review. J. Environ. Chem. Eng..

[8] He S., Li H., Shen T., Sun J., Pan H., Sun X., Lu W., Lu X., Gao G., Fan Y. (2023). Preparation and performance of multi-ionic composite coagulants based on coal gasification coarse slag by one-step acid leaching. Process Saf. Environ. Protect..

[9] Yuan N., Zhao A., Hu Z., Tan K., Zhang J. (2021). Preparation and application of porous materials from coal gasification slag for wastewater treatment: A review. Chemosphere.

[10] Yang X., Tang W., Liu X., Du H., Wu Y., Zhang J. (2019). Synthesis of mesoporous silica from coal slag and co_2_ for phenol removal. J. Clean. Prod..

[11] Velty A., Corma A. (2023). Advanced zeolite and ordered mesoporous silica-based catalysts for the conversion of co_2_ to chemicals and fuels. Chem. Soc. Rev..

[12] Yokoi T., Kubota Y., Tatsumi T. (2012). Amino-functionalized mesoporous silica as base catalyst and adsorbent. Appl. Catal. A Gen..

[13] Ncube T., Kumar Reddy K.S., Al Shoaibi A., Srinivasakannan C. (2017). Benzene, toluene, *m*-xylene adsorption on silica-based adsorbents. Energy Fuels.

[14] Qi P., Xu Z., Zhou T., Zhang T., Zhao H. (2021). Study on a quartz crystal microbalance sensor based on chitosan-functionalized mesoporous silica for humidity detection. J. Colloid. Interface. Sci..

[15] Li C., Qiao X. (2016). A new approach to prepare mesoporous silica using coal fly ash. Chem. Eng. J..

[16] Zhu D., Zuo J., Jiang Y., Zhang J., Zhang J., Wei C. (2020). Carbon-silica mesoporous composite in situ prepared from coal gasification fine slag by acid leaching method and its application in nitrate removing. Sci. Total Environ..

[17] Liu S., Chen X., Ai W., Wei C. (2019). A new method to prepare mesoporous silica from coal gasification fine slag and its application in methylene blue adsorption. J. Clean. Prod..

[18] Xu Y., Ai W., Zuo J., Yang W., Wei C., Xu S. (2022). Mesoporous spherical silica filler prepared from coal gasification fine slag for styrene butadiene rubber reinforcement and promoting vulcanization. Polymers.

[19] Wei X., Liu J., Yan H., Li T., Wang Y., Zhao Y., Li G., Zhang G. (2025). Synthesis of large mesoporous silica for efficient CO_2_ adsorption using coal gasification fine slag. Sep. Purif. Technol..

[20] Shu Q., Sun Z., Zhu G., Wang C., Li H., Qi F., Zhang Q., Li S. (2022). Highly efficient synthesis of zsm-5 zeolite by one-step microwave using desilication solution of coal gasification coarse slag and its application to vocs adsorption. Process Saf. Environ. Protect..

[21] Chai Z., Liu B., Lv P., Bai Y., Wang J., Song X., Su W., Yu G. (2023). Recycling of coal gasification fine slag as ultra-high capacity adsorbents for the removal of rhodamine b dye: Graded synthesis method, kinetics and adsorption mechanism. Fuel.

[22] Liu B., Lv P., Wu R., Bai Y., Wang J., Su W., Song X., Yu G. (2023). Coal gasification fine slag based multifunctional nanoporous silica microspheres for synergistic adsorption of pb(ii) and congo red. Sep. Purif. Technol..

[23] Yang B., Han F., Li Y., Bai Y., Xie Z., Yang J., Liu T. (2023). Phosphate removal mechanism of a novel magnesium slag-modified coal gasification coarse slag adsorbent. Environ. Sci. Pollut. Res..

[24] Han Y., Qi W., Pang H., Zhao Q., Huang Y., Zhao D., Zhu W., Zhang J. (2024). A novel coal gasification coarse slag-based geopolymer: Influences of physico-chemical coupling activation on its properties, microstructure, and hazardous material immobilization. Constr. Build. Mater..

[25] Huang Y., Qiu Y., Zhang Z., Wang W., Peng W., Cao Y. (2024). Synthesis of silane-modified mesoporous silica from coal gasification coarse slag and its novel application on gallium extraction. Sep. Purif. Technol..

[26] Yan S., Xuan W., Cao C., Zhang J. (2023). A review of sustainable utilization and prospect of coal gasification slag. Environ. Res..

[27] Li T., He S., Shen T., Sun J., Sun C., Pan H., Yu D., Lu W., Li R., Zhang E. (2022). Using one-step acid leaching for the recovering of coal gasification fine slag as functional adsorbents: Preparation and performance. Int. J. Environ. Res. Public Health.

[28] Chen J., Wang W., Zhou L., Pan Z. (2021). Effect of Al_2_O_3_ and mgo on crystallization and structure of CaO–SiO_2_–B_2_O_3_-based fluorine-free mold flux. J. Iron Steel Res. Int..

[29] Peng L., Qisui W., Xi L., Chaocan Z. (2009). Investigation of the states of water and oh groups on the surface of silica. Colloids Surf. A Physicochem. Eng. Asp..

[30] Xu Y., Weng S. (2016). Fourier Transform Infrared Spectral Analysis.

[31] Banerjee P., Chakraborty T. (2021). Confinement effects on c–h and c–f stretching vibrational frequencies of difluoromethane in cold inert gas matrixes: A combined infrared spectroscopy and electronic structure theory study. Eur. Phys. J. D.

[32] Chen H., Sun Z.Y., Shao J. (2011). Infrared Spectral Characteristics of Silica from Eight Different Sources. Bull. Chin. Silic. Soc..

[33] Qin Z., Wang S., Fan L., Zhou C., Wang C., Song L., Ma K., Yue H. (2023). A hydrazine amidoxime crosslinked polyacrylonitrile resin for efficient extraction of gallium from vanadium-containing waste solution. Chem. Eng. Sci..

[34] Sontakke A.D., Annapurna K. (2012). Network coordination in low germanium alkaline-earth gallate systems: Influence on glass formation. RSC Adv..

[35] Wang Y., Zhu L., Song Y., Lou Z., Shan W., Xiong Y. (2020). Novel chitosan-based ions imprinted bio-adsorbent for enhanced adsorption of gallium(iii) in acidic solution. J. Mol. Liq..

[36] Kang S., Hong S.I., Choe C.R., Park M., Rim S., Kim J. (2001). Preparation and characterization of epoxy composites filled with functionalized nanosilica particles obtained via sol–gel process. Polymer.

[37] Lin O.H., Md Akil H., Ishak Z.A.M. (2009). Characterization and properties of activated nanosilica/polypropylene composites with coupling agents. Polym. Compos..

[38] Fujiki J., Yogo K., Furuya E. (2018). Role of silanol groups on silica gel on adsorption of benzothiophene and naphthalene. Fuel.

[39] Balcom H., Hoffman A.J., Locht H., Hibbitts D. (2024). Correction to “brønsted acid strength does not change for bulk and external sites of mfi except for al substitution where silanol groups form”. ACS Catal..

[40] Maximiano P., Simões P.N. (2024). Silica aerogel-carbon nanotube composites: Mechanistic insights into condensation reactions. Chem. Eng. J..

