# Supplementary material for: Enhanced Gallium Extraction Using Silane-Modified Mesoporous Silica Synthesized from Coal Gasification Slag
Source: Molecules. 2024 Nov 5;29(22):5232. doi: 10.3390/molecules29225232 (PMC11596242; doi:10.3390/molecules29225232)
Supplement: Supplementary file 1 [file molecules-29-05232-s001.zip › molecules-3286617-supplementary.pdf]

Supporting Information

For

Enhanced Gallium Extraction Using Silane-Modified Mesoporous Silica Synthesized from Coal Gasification Slag

Shiqiao Yang[a,1], Guixia Fan[a,b,c,1], Lukuan Ma[a], Peng Li[a,b,c,], Yijun Cao[a,b,c*], Daoguang Teng[a,b,c*]

[a] School of Chemical Engineering, Zhengzhou University, Zhengzhou, 450001, China

[b] Zhongyuan Critical Metals Laboratory, Zhengzhou, 450001, China

[c] The Key Lab of Critical Metals Minerals Supernormal Enrichment and Extraction, Ministry of Education, Zhengzhou, 450001, China

*Corresponding Author

Daoguang Teng: teng_daoguang@zzu.edu.cn

Experimental Section

1. Acid leaching experiments

1.1 Impact of Different Acids on CGCS Modification Experiment

CGCS (4 g) was added to an acid solution (H^+ (aq), 8 mol/L 30 mL), namely HCl (8 mol/L), H_2SO_4 (4 mol/L), and HAc (8 mol/L), separately in pressure-resistant bottles. The reactions were conducted under constant agitation in a temperature-controlled water bath at 90°C and 120 rpm for 8 h. Post-reaction, samples were rinsed with deionized water and filtered until the filtrate reached near-neutral pH. The acid-leached CGCS (H-CGCS) samples were then dried in a hot air oven for 12 h and analyzed using XRD, FTIR, SEM, and BET techniques. The acid-leached samples were named A-CGCS, where A denotes the acid used.

(Moving forward, the liquid-to-solid ratio will be 15:2.)

1.2 Effect of Acid Concentration on CGCS Modification Experiment

A total of 30 mL of hydrochloric acid at concentrations of 2, 4, 6, 8, 10, and 12 mol/L was individually reacted with 4 g of CGCS under constant agitation in a temperature-controlled water bath at 90°C and 120 rpm for 8 h. Post-reaction, samples were rinsed with deionized water and filtered until the filtrate reached near-neutral pH. The filter cake samples were dried in a hot air oven for 12 h and analyzed using BET. The samples were named HCl(C)-CGCS (C represents HCl concentration in mol/L).

1.3 Effect of Acid Leaching Time on CGCS Modification Experiment

Hydrochloric acid (6 mol/L 30 mL) was reacted with CGCS (4 g) under constant

agitation in a temperature-controlled water bath at 90°C and 120 rpm for 1 to 8 h. Post-reaction, samples were rinsed with deionized water and filtered until the filtrate reached near-neutral pH. The filter cake samples were dried in a hot air oven for 12 h and analyzed using BET. The samples were named HCl-CGCS-t(a) (a represents reaction time in h).

1.4 Effect of Acid Leaching Temperature on CGCS Modification Experiment

Hydrochloric acid (6 mol/L 30 mL) was reacted with CGCS (4 g) at temperatures ranging from 20 to 95°C under constant agitation in a temperature-controlled water bath for 3 h. Post-reaction, samples were rinsed with deionized water and filtered until the filtrate reached near-neutral pH. The filter cake samples were dried in a hot air oven for 12 h and analyzed using BET. The samples were named HCl-CGCS-T(b) (b represents reaction temperature in °C).

2 Adsorption Experiment

Using HCl(6)-CGCS-t(3)-T(90) as the adsorbent, the Ga(III) solution from the $GaCl_3$ source was adsorbed. A series of single-factor optimization experiments were conducted to investigate its adsorption kinetics, isotherms, and competitive adsorption in alkaline systems. Below is a detailed description of the experiments.

2.1 Solution pH

To prevent Ga solution precipitation, the Ga^{3+}(40 mg/L 50 mL) solution pH was adjusted to 1, 2, 3, 9, 10, 11, and 12 using NaOH and HCl solutions. Under constant shaking in a temperature-controlled shaking incubator at 30°C and 200 rpm, an

adsorbent (300 mg) was added to each pH condition for 3 h. The Ga ion concentrations before and after adsorption were analyzed using ICP. The adsorption percentage (A%) and adsorption capacity (qe, mg/g) were calculated according to Equations (S1) and (S2).

$$q_e = \frac{(c_0 - c_e)V}{m} \tag{S1}$$

$$A\% = \frac{c_0 - c_e}{c_0} \times 100\% \tag{S2}$$

In the equation, qe represents the equilibrium adsorption capacity (mg/g), A (%) denotes the adsorption percentage, c_0 and c_e, respectively, indicate the initial concentration of metal ions and the residual concentration of a specific ion in the solution after the reaction (mg/L), while V (mL) and m (mg) represent the volume of the Ga ion solution (mL) and the mass of the adsorbent (g).

2.2 Adsorbent Dosage

Six sets of adsorbents ranging from 100 to 550 mg were weighed. Each set was used to adsorb 50 mL of 40 mg/L Ga^{3+} solution, with the pH adjusted to the optimized value. The adsorption was conducted in a constant temperature shaking incubator at 30°C and 200 rpm for 3 h to determine the optimal adsorbent dosage.

2.3 Initial Solution Concentration

Several sets of 50 mL Ga^{3+} solutions with concentrations ranging from 20 to 150 mg/L were prepared. The pH was adjusted to the optimized value, and the optimized adsorbent dosage was used. Adsorption was conducted in a constant temperature shaking incubator at 30°C and 200 rpm for 3 h to determine the optimal initial

concentration of the Ga(III) solution.

2.4 Adsorption Time

Under the optimized conditions, adsorption was conducted for 3 h to determine the equilibrium adsorption time.

2.5 Adsorption Kinetics

(1) Pseudo First-Order (PFO) Kinetic Model

The PFO model suggests that the rate-limiting step during adsorption is primarily diffusion. This model is generally applicable to adsorption processes with the following characteristics: (1) a high initial concentration C_0; (2) early stages of the adsorption process, where the PFO model approximates the pseudo second-order (PSO) model as time t approaches zero, and can well describe the adsorption behavior in the first 20 minutes of kinetic data; and (3) a limited number of active sites on the adsorbent, making external or internal diffusion the rate-limiting step. The PFO model is expressed as follows in Equation (S3):

$$\ln(Q_e - Q_t) = \ln Q_e - K_1 t \quad \text{(S3)}$$

(2) Pseudo Second-Order (PSO) Kinetic Model

The PSO kinetic model reflects an adsorption mechanism dominated by chemisorption. Research has established the physical meaning of the PSO model. Generally, adsorption processes suitable for the PSO model exhibit the following characteristics: (1) a low initial concentration; (2) adsorption occurring in the later stages of the process; and (3) an abundance of active sites on the adsorbent. The PSO

model is described by Equation (S4).

$$\frac{t}{Q_t}=\frac{1}{K_2Q_e^2}+\frac{t}{Q_e} \tag{S4}$$

where Q_e and Q_t (mg/g) represent the adsorption capacity per unit of adsorbent at equilibrium and at time t (min), respectively. K_1 (min^{-1}) and K_2 (g/mg·min) are the rate constants of the pseudo first-order and pseudo second-order kinetic models, respectively.

2.6 Adsorption Isotherms

Adsorption isotherms describe the relationship between the equilibrium concentration of the adsorbate in a solution and the amount of adsorbate adsorbed onto the solid phase at a constant temperature. This relationship not only reveals the interactions between the adsorbent and adsorbate but also reflects the surface properties and affinity of the adsorbent. By modeling and fitting the equilibrium adsorption data to isotherm models, valuable insights can be gained into the adsorption mechanism, maximum adsorption capacity, and characteristics of the adsorbent. The adsorption models used in this study include the Langmuir and Freundlich isotherm models.

(1) Langmuir Isotherm Model

The Langmuir isotherm model is based on several key assumptions, namely that all adsorption sites have equal adsorption probability (i.e., the surface is homogeneous); adsorption occurs as a monolayer; and adsorption at one site is independent of adsorption at neighboring sites. The model is based on the concept of an idealized adsorption layer, and its isotherm equation is expressed as follows in Equation (S5):

$$\frac{C_e}{Q_e}=\frac{1}{Q_m b}+\frac{C_e}{Q_m} \quad \text{(S5)}$$

where Q_e (mg/g) is the amount of adsorbate adsorbed at equilibrium; C_e(mg/L) is the equilibrium concentration of Ga(III); Q_m (mg/g) is the maximum adsorption capacity; and b is the Langmuir constant related to the affinity of binding sites. The dimensionless separation factor R_L, which indicates the feasibility of adsorption, is expressed as follows in Equation (S6):

$$R_L=\frac{1}{1+bC_0} \quad \text{(S6)}$$

where C_0（mg/L）is the initial concentration of Ga(III). When R_L=0, the adsorption is irreversible; 0<R_L<1 indicates favorable adsorption; R_L=1 indicates linear adsorption; R_L>1 indicates unfavorable adsorption.

（2）Freundlich Isotherm Model

The Freundlich isotherm model takes into account the heterogeneous nature of most solid surfaces, assuming that adsorption occurs on a heterogeneous surface and thus represents non-ideal adsorption. The isotherm equation is expressed as follows in Equation (S7):

$$\ln Q_e=\ln K_F+\frac{\ln C_e}{n} \quad \text{(S7)}$$

where Q_e（mg/g）is the amount of adsorbate adsorbed at equilibrium; C_e（mg/L）is the equilibrium concentration of Ga(III); K_F(L/mg) is the Freundlich constant indicative of adsorption capacity; and 1/n (dimensionless) is the Freundlich constant indicative of adsorption intensity.

2.7 Competitive Adsorption

To investigate the adsorption selectivity of the adsorbent for Ga(III) at pH 9, it is essential to consider the presence of competing ions such as Al, V, and Ge, which are commonly found under alkaline conditions. Therefore, Al, V, and Ge were selected as competing ions. The selectivity of H-CGCS for Ga(III) was examined in binary systems of Al/Ga, V/Ga, and Ge/Ga, each with an ion concentration of 40 mg/L and a total volume of 50 mL. To illustrate the adsorbent's affinity for Ga(III) more clearly, the selectivity coefficient $Sel_{(Ga/M)}$ was calculated. The selectivity coefficient (Sel) is determined using Equation (S8).

$$\mathrm{Sel}_{\mathrm{Ga/M}} = \lg \frac{K_{d-\mathrm{Ga}}}{K_{d-M}} = \lg \frac{(Q_{e1} / C_{e1})_{\mathrm{Ga}}}{(Q_{e2} / C_{e2})_{\mathrm{M}}} \quad \text{(S8)}$$

where M is the competing ion, C_{e1} (mg/L) is the equilibrium concentration of Ga(III), C_{e2} (mg/L) is the equilibrium concentration of the competing ion, Q_{e1} (mg/L) is the adsorption amount of Ga(III) at equilibrium, and Q_{e2} (mg/L) is the adsorption amount of the competing ion at equilibrium.

Results and discussion

3.5. Regeneration cycle

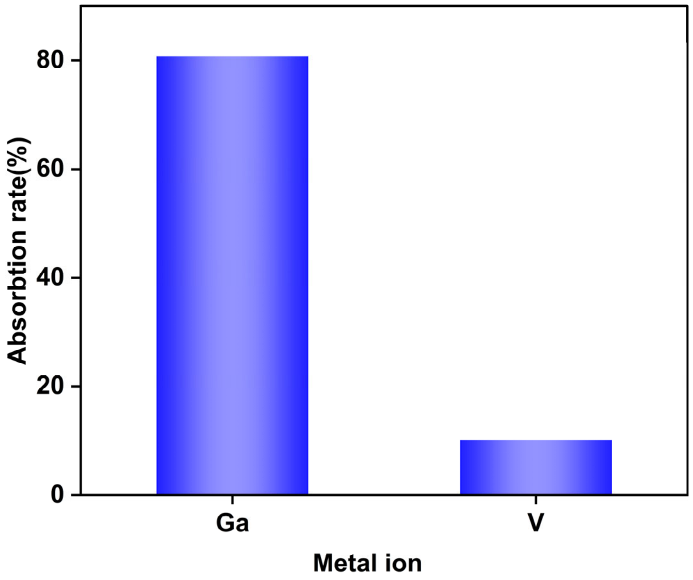

Figure S1 Competitive adsorption in the Ga-V binary system.

Table S1 Selectivity coefficients for Ga/V competitive adsorption.

System	$K_{d\text{-}Ga}$	$K_{d\text{-}V}$	$Sel_{Ga/V}$
Ga/V	0.4185	0.0112	1.5736
